# SiBIC: A Web Server for Generating Gene Set Networks Based on Biclusters Obtained by Maximal Frequent Itemset Mining

**DOI:** 10.1371/journal.pone.0082890

**Published:** 2013-12-30

**Authors:** Kei-ichiro Takahashi, Ichigaku Takigawa, Hiroshi Mamitsuka

**Affiliations:** 1 Bioinformatics Center, Institute for Chemical Research, Kyoto University, Uji, Kyoto, Japan; 2 Creative Research Institution, Hokkaido University, Sapporo, Hokkaido, Japan; Nazarbayev University, Kazakhstan

## Abstract

Detecting biclusters from expression data is useful, since biclusters are coexpressed genes under only part of all given experimental conditions. We present a software called SiBIC, which from a given expression dataset, first exhaustively enumerates biclusters, which are then merged into rather independent biclusters, which finally are used to generate gene set networks, in which a gene set assigned to one node has coexpressed genes. We evaluated each step of this procedure: 1) significance of the generated biclusters biologically and statistically, 2) biological quality of merged biclusters, and 3) biological significance of gene set networks. We emphasize that gene set networks, in which nodes are not genes but gene sets, can be more compact than usual gene networks, meaning that gene set networks are more comprehensible. SiBIC is available at http://utrecht.kuicr.kyoto-u.ac.jp:8080/miami/faces/index.jsp.

## Introduction

A biologically significant interest would be to detect genes with similar behavior under certain experimental conditions. SiBIC is a web server, which, given an expression dataset, provides such gene behavior information in a compact manner. The idea behind SiBIC is to enumerate all possible gene behaviors as biclusters, which are then summarized into gene set networks, in which each node has a gene set with coexpressed genes under particular experimental conditions. The procedure of SiBIC is as follows: SiBIC first enumerates all biclusters in a given expression dataset which are then merged together into a relatively smaller number of rather unique biclusters, from which finally gene set networks are generated. Gene set networks have a set of genes for nodes, by which each node can have more than one genes. Thus gene set networks are clearly more advantageous than usual gene networks, because the network size can be kept smaller while genes at each node are coexpressed.

**Figure 1:**
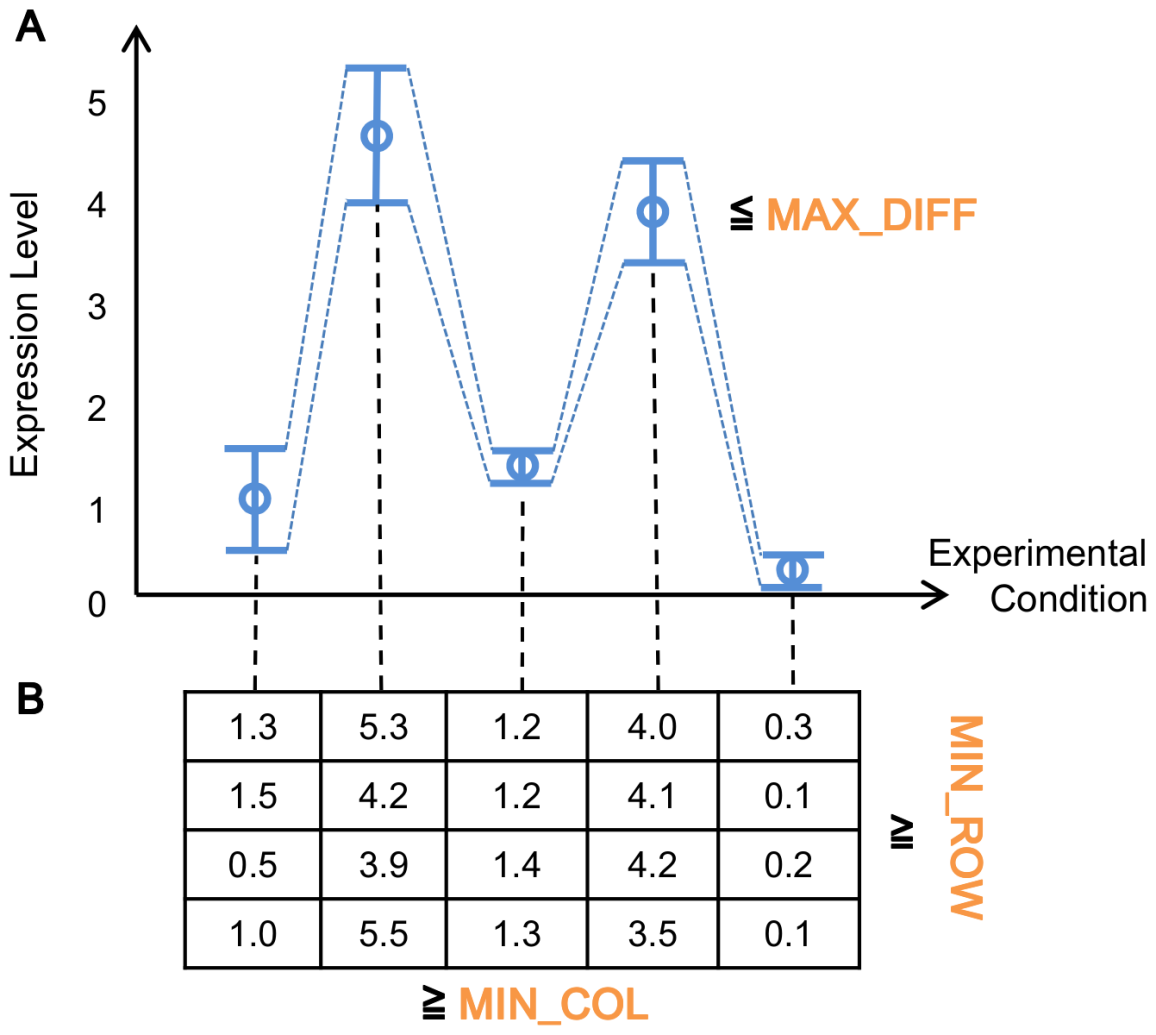
Biclusters of four coexpressed genes under five different conditions. Each bicluster can be defined by three parameters: MIN_ROW, MIN_COL and MAX_DIFF, where MIN_ROW is the minimum number of rows, MIN_COL is the minimum number of columns and MAX_DIFF is the maximum difference in values of each column.

**Figure 2:**
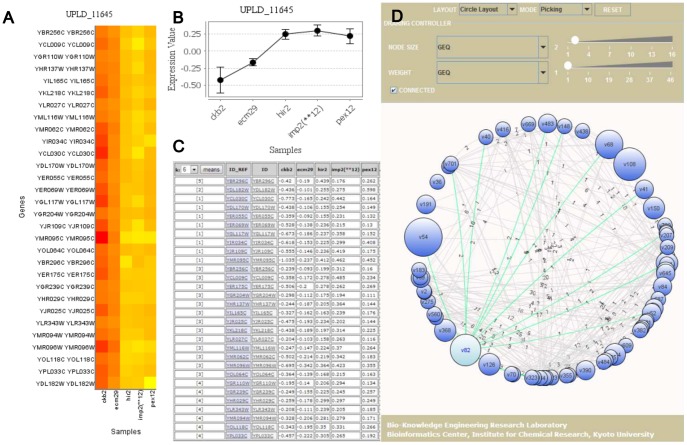
A found bicluster is shown in three formats and overlapping biclusters are converted into a gene set network. (A) a heat map, (B) a line chart, (C) a matrix of expression values, and (D) a sample gene set network, where nodes are clickable to show the corresponding genes or biclusters.

Biclusters can be classified into several different types [1], [2]. We focus on one type, in which genes are coexpressed under each experimental condition. Fig. 1 shows an example of such biclusters, where values are similar in each column. More concretely values in the first column are around one, while those in the second column are around four to five. This bicluster reveals genes which behave similarly under certain experimental conditions, and so finding such biclusters from a given expression data set can help capturing such genes. To enumerate this type of biclusters exhaustively from a given expression dataset, SiBIC uses *frequent itemset mining* (FIM), a well-established data mining technique [3]. In data preprocessing, for each experimental condition, SiBIC first generates items, each having one or more genes with similar expression values. This process transforms a given expression data matrix into a new matrix, where each element (originally a gene expression value) is a set of items. SiBIC then tries to find a set of items, i.e. an itemset, in which each item is from one experimental condition (i.e. one column) and all these items share the same set of genes. In particular, by using the idea of FIM, SiBIC enumerates all itemsets, the number of items being larger than a certain amount, and those itemsets are all biclusters, each having genes with similar expression values under each condition. One problem of these biclusters is that they are heavily overlapped and redundant. Thus SiBIC merges biclusters if the significance of biclusters is kept or improved. The first output of SiBIC is these biclusters, being sorted by size or *p*-values regarding the correlation significance of coexpression values. Fig. 2 (A)–(C) are sample outputs of biclusters, where (A) is a heat map with denser red for lower values, (B) is a chart showing the medium and min-max range of each column, and (C) a matrix of real expression values. However, the number of merged biclusters is still large, and then SiBIC presents gene set networks, where nodes are overlapped subclusters of the merged biclusters. Fig. 2 (D) is a sample output of gene set networks. SiBIC further allows to conduct GO (Gene Ontology) term enrichment analysis of each merged bicluster by using DAVID [4].

There are many software for generating biclusters, while the definition of biclusters is diverse, resulting in that the objective of most bicluster software is different from SiBIC, by which they cannot be necessarily compared with SiBIC. We here raise several software for generating/visualizing biclusters which can be compared with SiBIC, and describe how SiBIC is different from them.

DeBi [5] and BiModule [6] are two existing tools of using FIM to generate biclusters from a gene expression dataset. A clear difference of SiBIC from DeBi and BiModule is that they generate a very small number, say five or seven, of items, by which expression values in one item are not necessarily so consistent. On the other hand, SiBIC generates as many items as possible, satisfying the input range of coexpression values, by which biclusters of SiBIC surely capture coexpressed genes. Moreover SiBIC generates a network of gene sets as well as biclusters, and this type of summary information cannot be provided by DeBi and BiModule.

BicAT [7] and BiVisu [8] are well-known visualization software on biclusters. They however focus on visualization of biclusters themselves rather than the relations between biclusters. They further assume that biclusters are obtained by other methods or generating biclusters using existing methods. A software, which might be more related with SiBIC is BicOverlapper [9], which is for visualization of overlapping biclusters, while this software generates a graph, in which each node represents a gene or a condition and edges are grouped by one or more biclusters. So BicOverlapper shows each bicluster as a complete graph in the whole graph. On the other hand, SiBIC uses another graph on biclusters, where nodes are gene sets. This enables the graph to be more compact than that of BicOverlapper, because each node is a set of genes (which share a similar expression pattern), instead of a single gene or a single condition.

We evaluated SiBIC from a variety of viewpoints. We first compared the performance of SiBIC with two FIM-based biclustering methods, DeBi and BiModule, in terms of GO term enrichment analysis and correlation of coexpression values, by using major two benchmark datasets. SiBIC clearly outperformed the two compared methods in the two datasets. We then evaluated our procedure of merging biclusters by using a dataset in GEO, comparing with the original, unmerged biclusters, in terms of GO term enrichment analysis. The result showed that merged biclusters were enhanced more than unmerged biclusters, confirming the validity of merging biclusters. We finally checked the validity of generating gene set networks. Again GO term enrichment analysis was conducted over 1) densely connected subnetworks and 2) the subnetwork containing the hub gene and neighboring genes, both results revealing the validity of representing gene set networks.

## Results and Discussion

We have evaluated each of the main three steps of SiBIC: 1) generating biclusters, 2) merging biclusters, and 3) generating gene set networks.

### Evaluation on biclusters

We evaluated the performance of biclusters generated by SiBIC with respect to two aspects: biological and statistical significance. The performance was compared with two methods, DeBi [5] and BiModule [6], which are based on FIM, and the performance advantage of these two methods over a number of representative biclustering algorithms such as Bimax [10], OPSM [11], ISA [12] and SAMBA [13] are already shown.

For biological and statistical evaluation, we conducted GO term enrichment analysis and empirical *p*-value computation on the correlation of row vectors of biclusters, respectively. These evaluation using a human and a yeast dataset have confirmed that 1) biclusters by SiBIC are enriched by GO terms more significantly than those by DeBi and BiModule and 2) correlation in row vectors of biclusters by SiBIC was more significant than those by DeBi and BiModule.

#### Biological significance: GO term enrichment analysis

Coexpressed genes in one bicluster are supposed to be all functioning on related regulatory mechanisms. For example, coexpressed genes may be controlled by the same transcription factors or involved in the same biological pathways. That is, an obtained bicluster should be connected to some biological function, by which genes in a bicluster must be well-enriched by some GO terms. GO term enrichment analysis is commonly used in evaluation of biclustering methods [10]. For example, DeBi [5] outperformed five methods: Bimax [10], OPSM [11], ISA [12], SAMBA [13] and QUBIC [14] in GO term enrichment analysis on a yeast dataset. Similarly BiModule [6] outperformed six methods: Bimax, OPSM, ISA, SAMBA, CC [15] and xMotif [16] on another yeast dataset. These results indicate that if SiBIC outperforms DeBi and BiModule, SiBIC can outperform totally seven methods for detecting biclusters.

To compare SiBIC with DeBi and BiModule, we used two datasets. The first dataset, which was used in [5], is the compendium of gene expression profiles with 300 different experimental conditions of *S. cerevisiae*. We ran SiBIC with the default parameter settings: MIN_ROW = 30, MIN_COL = 3, BIN = 7 and SD_COEFF = 0.7. BiModule was executed with Mg = 30, Mc = 3 and L = 7, where Mg, Mc and L correspond to MIN_ROW, MIN_COL and BIN, respectively. As for DeBi, we used the resultant biclusters given in [5] which were obtained by running DeBi over the same yeast data set. In order to make a fair evaluation, we applied a similar procedure of DeBi to the resultant clusters of SiBIC and BiModule. That is, the procedure is to filter out biclusters being overlapped with other biclusters. More concretely, if more than 50% of one bicluster is overlapped with another larger bicluster, we removed the smaller cluster. We finally selected top 100 biclusters by their size for each method.

**Figure 3:**
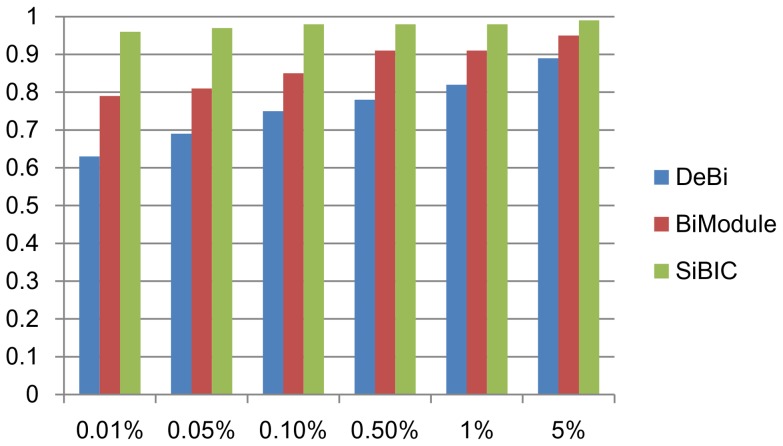
GO term enrichment analysis for biclusters generated by a yeast dataset. SiBIC outperformed DeBi and BiModule at any significance level from 0.01% to 5%.

We used a GO term enrichment tool, FuncAssociate, which computes the *p*-value of how significantly one gene set is enriched by one or more GO terms, using Fisher's exact test and multiple testing correction [17]. Figure 3 shows the ratio of biclusters enriched by at least one GO terms to all 100 biclusters at different levels of significance. SiBIC achieved the best performance among the compared three methods at each level of significance. In particular it is noteworthy to raise that 96% of biclusters by SiBIC were enriched by at least one GO terms at the significance level of 0.01%.

**Figure 4:**
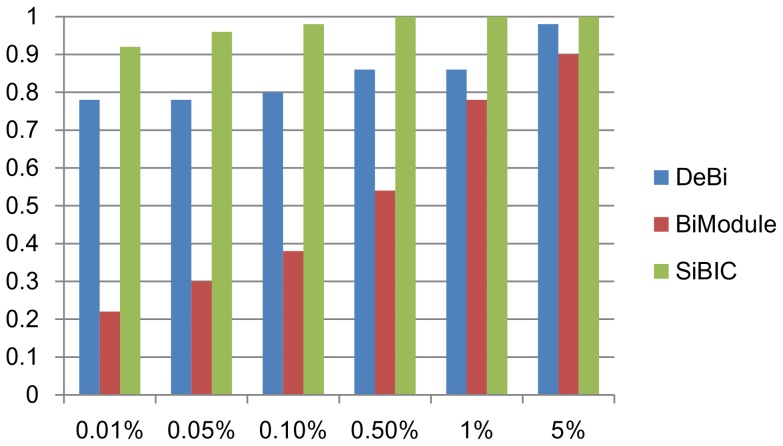
GO term enrichment analysis for biclusters generated by a human dataset. Again SiBIC outperformed DeBi and BiModule at all significance levels from 0.01% to 5%.

The second dataset is a human dataset, ‘diffuse large B-cell lymphoma’ (DLBCL), which is also assessed in [5]. DLBCL has 661 genes and 180 conditions. We ran SiBIC with MIN_ROW = 20, MIN_COL = 3, BIN = 7 and SD_COEFF = 0.7. BiModule was executed with Mg = 10, Mc = 3 and L = 7 (because BiModule could not produce any biclusters with Mg = 20). For DeBi, we again used the experimental results in [5]. Again we removed overlapped clusters, as done for the yeast dataset in the same way, resulting in 4,350, 192 and 53 biclusters, for SiBIC, DeBi and BiModule, respectively. We then selected top 50 biclusters by size for each of the three methods. Figure 4 shows the ratio of biclusters enriched by at least one GO terms to all 50 biclusters at different significance levels. This result clearly indicates that SiBIC generates a larger number of biclusters enriched by GO terms than those by DeBi and BiModule.

#### Statistical significance

We checked the statistical significance of each bicluster by how significantly row vectors (genes) in each bicluster are correlated with each other. We computed the significance score (empirical 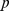-value) by using test statistic 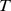 in Eq. (1) which will be described in the Method and Materials section. We used two experimental datasets, yeast and human (DLBCL), which were used for checking biological significance. Also we kept the same parameter setting as in the GO term enrichment analysis.

**Figure 5:**
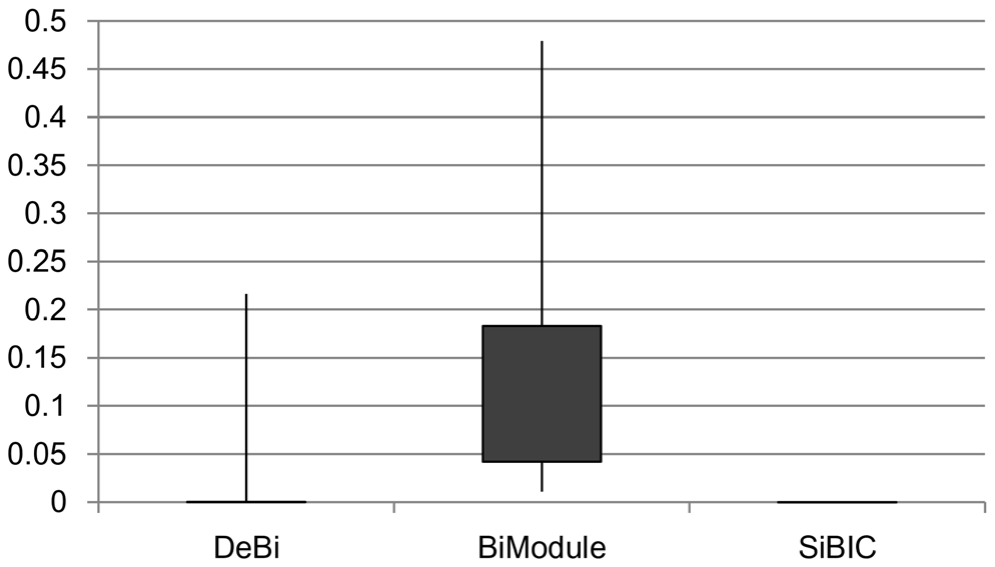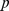-values of biclusters from a yeast dataset. The results clearly show that 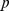-values by SiBIC are all almost zero, while the distribution of those of DeBi ranges from zero to 0.2 and that of BiModule was further higher.

Fig. 5 shows the box and whisker charts, showing the distribution of empirical 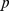-values for yeast. We computed empirical 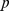-values for the top 100 biclusters by size for each method. Each black box in Fig. 5 indicates the 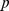-values of the top 25 to 75 of 100 biclusters. This figure shows that all 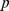-values by SiBIC were almost zero, by which biclusters by SiBIC had the distribution of clearly lower 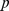-values than DeBi and BiModule.

**Figure 6:**
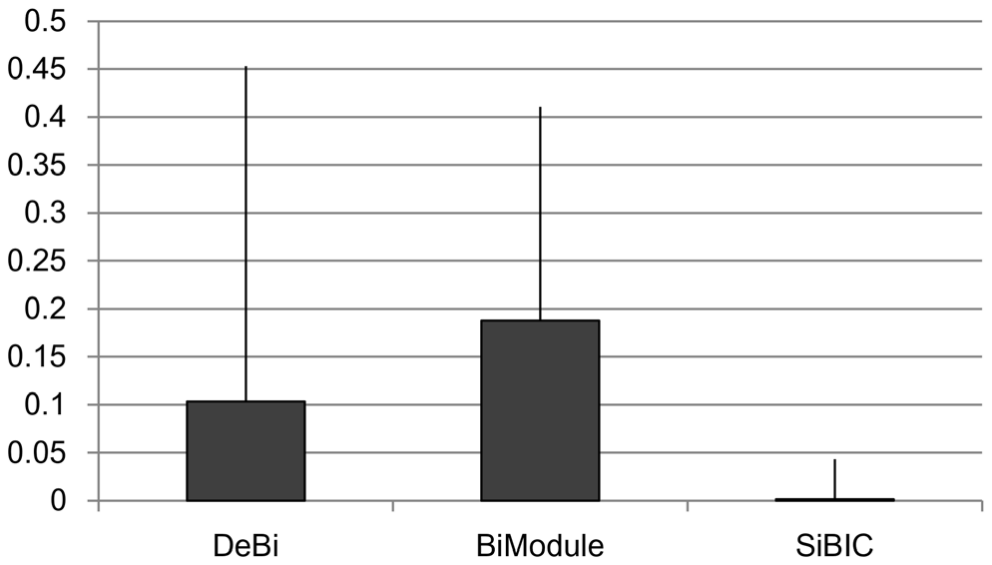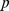-values of biclusters from a human dataset. The 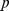-values of SiBIC were in the range from zero to 0.05, while those of DeBI and BiModule were distributed in wider ranges, including 0.1 to 0.4.

Fig. 6 shows the box and whisker charts of empirical 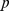-values for human (DLBCL). This figure shows that all 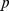-values by SiBIC were less than 0.05, while most 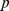-values of the other two methods were larger than 0.05, indicating that 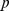-values by SiBIC were clearly smaller than those by DeBi and BiModule.

These results indicate that SiBIC can generate biclusters with the most significant correlation in gene coexpression among the three competing methods. This is because: 1) BiModule generates only seven items per column, which is too coarse to capture gene coexpression, and 2) Similarly DeBi transforms expression values into only three types, which is also too coarse, making hard to capture gene coexpression for each column.

#### Performance variation by parameter setting

**Figure 7:**
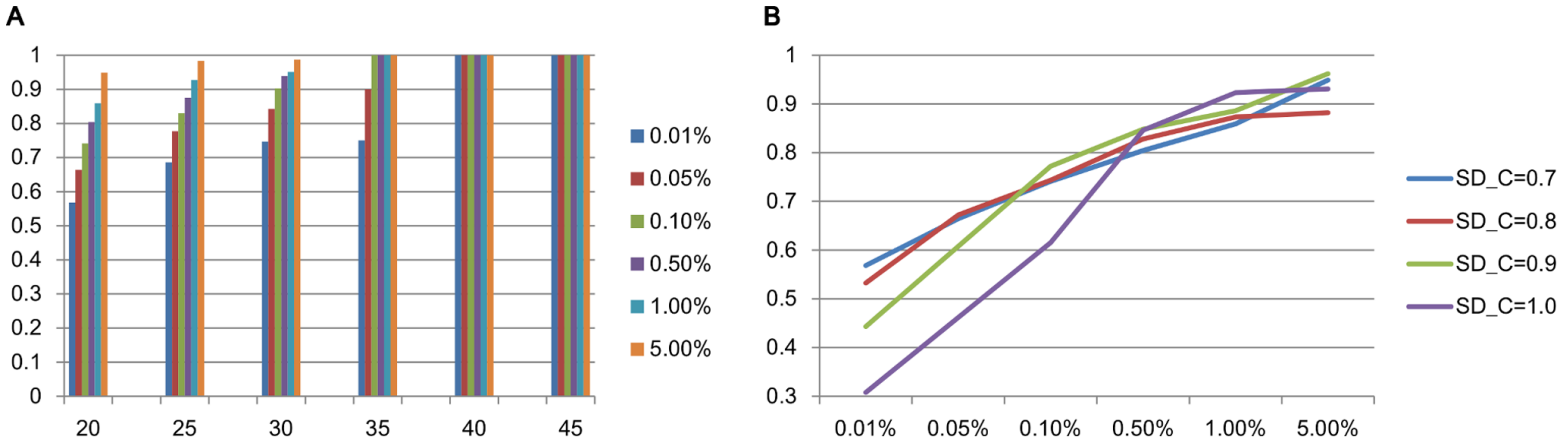
Performance variation in GO term enrichment analysis by changing values of parameters, (A) MIN_ROW and (B) SD_COEFF. In (A), blue, red, green, purpose, light blue and orange show the ratio of enriched biclusters to all biclusters at the significance level of 0.01%, 0.05%, 0.1%, 0.5%, 1% and 5%, respectively. The values along with the X-axis are the parameter values of MIN_ROW. In (B), blue, red, green and purple show the ratio of enriched biclusters to all biclusters at SD_COEFF = 0.7, 0.8, 0.9 and 1.0, respectively. The values along with the X-axis are the significance level of 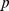-values.

We further checked the performance variation of SiBIC by changing values of parameters, particularly MIN_ROW and SD_COEFF, using DLBCL. The parameter setting we used so far was MIN_ROW = 20 (or 30) and SD_COEFF = 0.7, which generates a relatively large number of biclusters, and if we decrease these values, we have a further larger number of biclusters (in fact 23,774 and 16,426 biclusters obtained when MIN_ROW = 15 and SD_COEFF = 0.6, respectively, even after biclusters are merged together, removing overlapped ones), for all of which we cannot run any GO enrichment analysis tool. Thus we checked the performance of SiBIC when the values of MIN_ROW and SD_COEFF were larger, meaning that the number of biclusters was decreased (See Tables 1 and 2 for the number of biclusters, respectively). We note that in this evaluation, we removed overlapped biclusters, according to the procedure we mentioned already. Fig. 7 (A) and (B) show the results obtained by increasing the value, i.e. 20 to 45 for MIN_ROW and 0.7 to 1.0 for SD_COEFF, respectively. These two figures indicate that as increasing with the parameter value, the ratio of biclusters enriched by GO terms to all biclusters increased.

**Table 1:** The number of maximum frequent itemsets (MFI) and the number of merged biclusters (MB) when we changed MIN_ROW from 15 to 45.

**MIN_ROW**	**15**	**20**	**25**	**30**	**35**	**40**	**45**
# MFI	993,757	236,314	67,151	22,255	8,874	4,380	2,760
# MB	23,774	4,350	760	160	30	2	3

**Table 2:** The number of maximum frequent itemsets (MFI) and the number of merged biclusters (MB) when we changed SD_COEFF from 0.6 to 1.0.

**SD_COEFF**	**0.6**	**0.7**	**0.8**	**0.9**	**1.0**
# MFI	803,634	236,314	64,062	18,692	6,458
# MB	16,426	4,350	704	170	26

### Evaluation on merging biclusters

We used GDS3513 in GEO (Gene Expression Omnibus) which is a human dataset of embryonic stem cell-derived cardiomyocytes. Under appropriate conditions, vivo embryonic stem cells are supposed to be differentiated into beating cardiomyocytes via an embryoid body intermediate. This dataset is measured to obtain insight into the mechanism underlying the differentiation of embryonic stem cells into cardiomyocytes. GDS3513 has 16 samples of 45,220 probes with four cell types. We used the average values over sample replicates.

We run SiBIC over this dataset under the parameter settings of MIN_ROW = 10, BIN = 7, MIN_COL = 3, SD_COEFF = 1.0 and ALPHA = 0.01, resulting in 474 biclusters, which were then merged into 53 significant biclusters.

**Figure 8:**
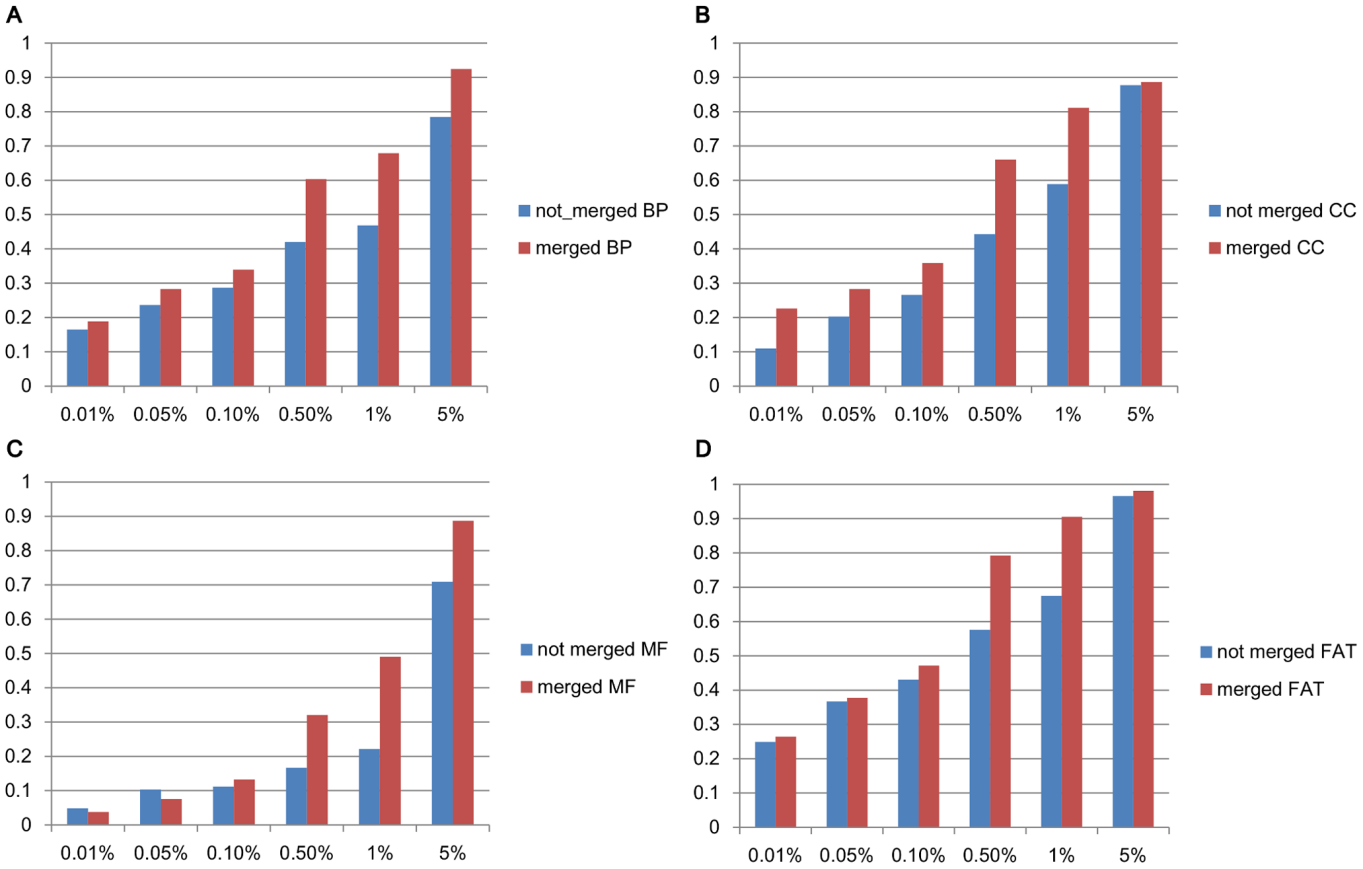
Comparison in terms of GO term enrichment analysis between merged and unmerged (original) biclusters. (A) BP (biological process), (B) CC (cell component), (C) MF (molecular function), (D) FAT (lower level GO terms).

In order to assess the effect of merging biclusters, we conducted GO term enrichment analysis using a software called DAVID, which allows to check the enrichment of coarser-level (upper-level) biological categories (than that of FuncAssociate), such as biological process terms (BP), molecular function terms (MF) and cellular component terms (CC), by which biclusters can be evaluated with respect to the entire, upper-level categories. Fig. 8 shows the ratio of enriched biclusters to all obtained biclusters for two cases: the original all enumerated biclusters and merged biclusters, for each of the three upper level functions (A)–(C) and usual, lower-level GO terms (called FAT) (D). For all four cases, the ratio of enriched biclusters was larger than the case of original biclusters for all significance levels. This result shows that merging biclusters can reduce redundant biclusters, without losing biological qualities of the obtained biclusters.

### Evaluation on gene set networks

**Figure 9:**
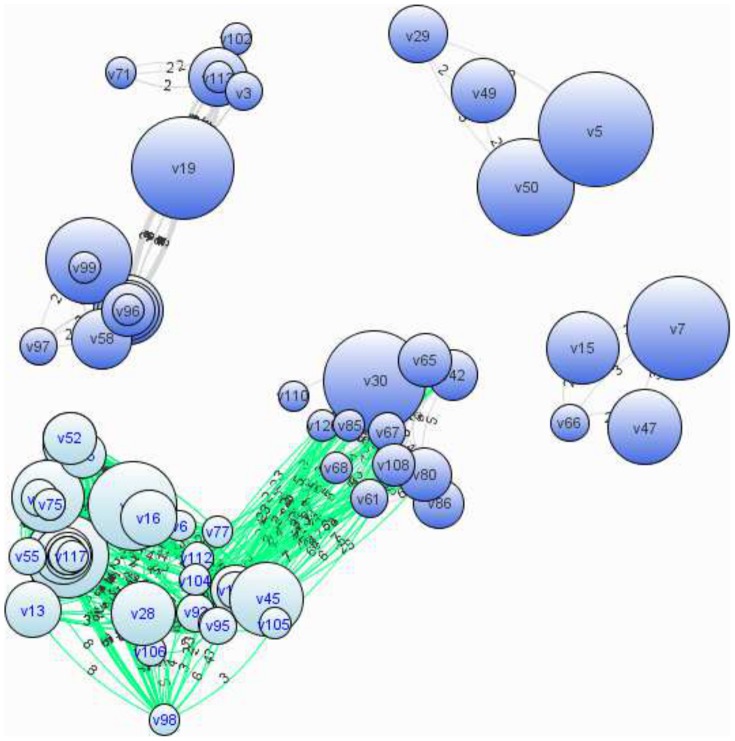
Four gene set networks obtained from GDS3513. Each node represents a gene or a set of genes. The size of a node represents the number of genes in the corresponding node.

In order to assess the quality of generated gene set networks, we used the same dataset as that used for evaluating merging biclusters. We first obtained four connected gene set networks from the 53 merged biclusters. Fig. 9 shows the resultant four networks.

We then conducted two types of GO term enrichment analysis on: 1) part of gene set networks with weights of more than or equal to two, and 2) part of gene set networks with the hub and connected nodes.

**Figure 10:**
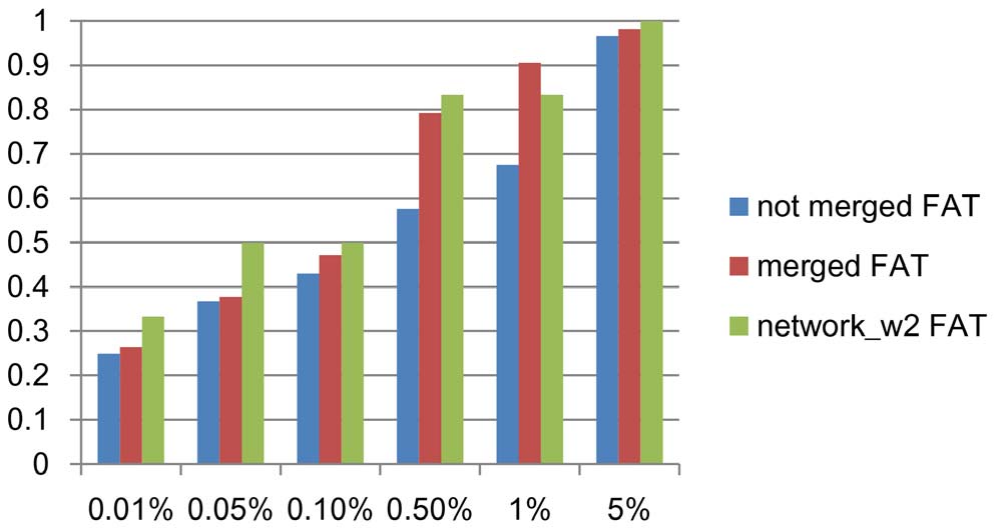
Comparison in terms of GO term enrichment analysis among original biclusters, merged biclusters and gene set networks.

In the first experiment, our goal is to check the beneficial of network representation. In order to do this, we focused on edges with weights of more than or equal to two, by which six gene set networks were obtained. We then run DAVID again over the six networks to perform GO term enrichment analysis. Fig. 10 shows the ratio of biclusters (or networks) which has at least one GO terms to all biclusters (or networks), changing the significance level. This figure shows that already half of the six networks were enriched by GO terms at the significance level of 0.05% and all six networks were covered at the significance level of 5%. From this result, we can say that gene set networks can represent the connection of biclusters (or gene sets) well enough, keeping the biological quality of the obtained information.

In the second experiment, we first chose the largest network, which consists of 44 overlapped biclusters with 128 nodes and 735 edges, including 1,761 genes in total. We can expect that biclusters, which have the maximally connected node, i.e. the node with the largest degree, must have the most important role in the whole network. The node with the largest degree was a gene set with only one gene, COL2A1 (collagen, type II, alpha 1), which is known as a gene related to heart morphogenesis. There were 46 nodes, which are all adjacent to COL2A1, under the condition that the edge weight is more than or equal to two. These 46 nodes have totally 156 genes. Fig. 9 shows these 46 nodes in the lower-left side. We enriched the 157 genes (156 genes plus COL2A1) by using DAVID, focusing on the category of biological process (BP), and obtained 125 BP terms. Table 3 shows the top 20 BP terms with the lowest 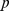-values. GO terms in this table include several terms, such as ‘heart’, ‘cardio’ and ‘cardiac’, which are all closely related with cardiomyocytes, i.e. the main topic of the dataset. In particular the top GO term with the lowest 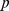-value of 8.32E-06 was heart development.

**Table 3:** Top 20 GO terms obtained by GO term enrichment analysis over genes in a hub network.

Rank	GO.ID	GO.term	P.value
1	GO:0007507	heart development	8.32E-06
2	GO:0003012	muscle system process	5.88E-05
3	GO:0045664	regulation of neuron differentiation	9.26E-05
4	GO:0003013	circulatory system process	1.20E-04
5	GO:0008015	blood circulation	1.20E-04
6	GO:0051960	regulation of nervous system development	1.49E-04
7	GO:0006936	muscle contraction	2.21E-04
8	GO:0003007	heart morphogenesis	2.28E-04
9	GO:0050767	regulation of neurogenesis	3.64E-04
10	GO:0048568	embryonic organ development	4.51E-04
11	GO:0042127	regulation of cell proliferation	4.92E-04
12	GO:0048562	embryonic organ morphogenesis	6.80E-04
13	GO:0048598	embryonic morphogenesis	7.77E-04
14	GO:0001755	neural crest cell migration	7.80E-04
15	GO:0035150	regulation of tube size	8.33E-04
16	GO:0050880	regulation of blood vessel size	8.33E-04
17	GO:0048704	embryonic skeletal system morphogenesis	0.001096
18	GO:0003018	vascular process in circulatory system	0.00117
19	GO:0008217	regulation of blood pressure	0.001219
20	GO:0060284	regulation of cell development	0.001269

We finally conducted an experiment of mapping a gene set network over metabolic pathways and checked whether the obtained pathways are related with each other. We focused on a relatively small network among the four connected network, since genes in such a small network might be all closely related with some particular function or pathway. So we used a network with 44 nodes, and extracted the node with the maximal degree and its neighboring nodes which are connected by edges with weights of more than or equal to two. The node with the maximal degree has only one gene, DDX43 (DEAD box polypeptide 43) encoding an ATP-dependent RNA helicase in the DEAD-box family. We then mapped the genes in these nodes over the KEGG pathways by using DAVID functional mapping tool [4]. Table 4 shows the pathways with 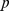-values of less than 0.05. This table shows that the pathways obtained are related with cell proliferation, differentiation and apoptosis, each other, which implies validity of the obtained pathways and a given gene set network.

**Table 4:** Pathways with *p*-values of less than 0.05, obtained by mapping a gene set network to KEGG by using DAVID functional mapping tool.

pathways	#genes	*p*-value
Cytokine-cytokine receptor interaction	17	1.3E-7
NOD-like receptor signaling pathway	8	1.3E-5
Hematopoietic cell lineage	5	4.9E-3
Apoptosis	6	5.1E-3
Cell adhesion molecules	6	6.4E-3
Intestinal immune network for IgA production	4	2.4E-2
Toll-like receptor signaling pathway	5	3.9E-2

### Conclusion

We have presented our software, SiBIC, which generates gene set networks by summarizing biclusters, which are first exhaustively enumerated based on maximal frequent itemset mining. We emphasize that gene set networks are more compact and comprehensible than usual gene networks, because each node has a set of coexpressed genes, by which the network size can be reduced. We evaluated each of our three steps of generating gene set networks: 1) enumerating biclusters, 2) merging biclusters and 3) generating gene set networks, mainly by using GO term enrichment analysis. Our evaluation results revealed that 1) our enumerated biclusters are biologically and statistically more significant than the compared two methods, 2) merging biclusters reduces the number of biclusters significantly, keeping the biological quality of the entire biclusters and 3) gene set networks are generated from merged biclusters, realizing compact representation of gene sets and at the same time again keeping/improving the biological quality. Overall SiBIC presents compact and comprehensible gene set networks which would be surely useful to biologically understand gene expression data.

## Materials and Methods

**Figure 11:**
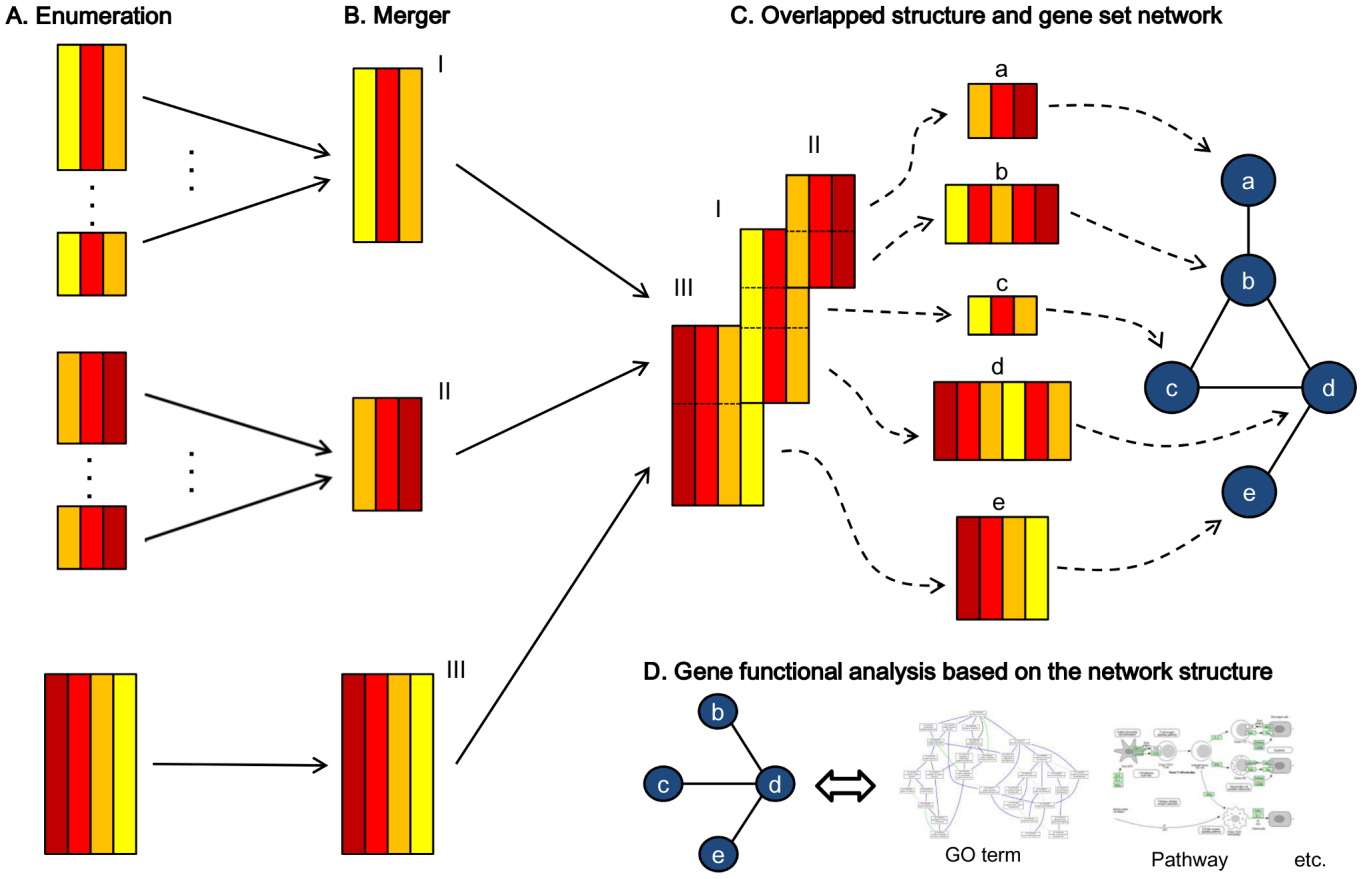
A flow diagram of SiBIC. (A) Enumerating all biclusters exhaustively by mining maximal frequent itemsets. Insignificant biclusters are filtered out by empirical hypothesis testing. (B) Merging overlapping biclusters with the same experimental conditions if they keep significance. (C) Generating gene set networks from overlapping biclusters. (D) Analyzing gene functions by using the obtained networks.

SiBIC has roughly five steps: 0) transforming a given original expression dataset into a matrix of itemsets, 1) enumerating all biclusters as frequent itemsets by using the idea of mining maximal frequent itemsets, 2) merging biclusters to remove redundancy in exhaustively enumerated biclusters, 3) generating a network of gene sets which are those overlapped among merged biclusters and 4) analyzing gene functions by using the generated network of gene sets. Figure 11 A) to D) show a schematic flow of the above 1) to 4), respectively.

### Data preprocessing and mining frequent itemsets

1. Data preprocessing (generating items): Given a gene expression matrix, we first generate items for each column (experimental condition). That is, for one column, we slide a window with a certain range (specified by MAX_DIFF. See below) over genes, which are already sorted by their expression values, meaning that genes in a window have similar expression values. A set of genes in each window is one item, by which one gene can be in multiple items. Repeating this over all experimental conditions (columns), the input matrix can be another matrix (of the same size), in which each element (originally gene) is a set of items. We note that items in one column are totally independent of those in another column.
2. Mining frequent itemsets: Out of the generated new matrix, the idea is to find a set of items (itemset) over multiple columns, where each item is from a different column and the same genes are shared by these items. This itemset is exactly a bicluster, since genes in this bicluster have similar expression values (because of an item) under each condition. A *frequent itemset* is a bicluster in which the number of contained genes is larger than or equal to a certain input parameter value (specified by MIN_ROW. See below). A *maximal frequent itemset* is the largest frequent itemset among all itemsets which hold inclusive relations each other (See below).

### Parameters in preprocessing and mining

SiBIC captures a particular type of biclusters, in which genes are regulated coherently in the same directions (up or down) with different magnitudes under specific experimental conditions. To define this type of biclusters, we use the following parameters, partially shown in Fig. 1.

1. MAX_DIFF: This parameter specifies the range of expression values of one item (Fig. 1A). We note that MAX_DIFF varies from one condition to another condition depending on the distributions of expression values. MAX_DIFF is not a direct input of SiBIC, and instead BIN is an input to compute MAX_DIFF by MAX_DIFF = (MAX
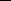
MIN)/BIN, where MAX and MIN are the maximum and minimum expression values in each column, respectively.
2. MIN_COL and MIN_ROW: MIN_COL and MIN_ROW are the minimum number of columns (experimental conditions) and rows (genes) of biclusters to be outputted, respectively. That is, out of the generated new item matrix, we retrieve a submatrix with a larger number of columns than MIN_COL and a larger number of rows than MIN_ROW. SiBIC exhaustively detects those biclusters which can be arbitrary positioned and overlapped in the input expression matrix. MIN_ROW specifies the minimum number of genes, which is important, while this can be relaxed, by merging biclusters which generates larger biclusters.
3. SD_COEFF: This parameter is used to remove expression values (of each column) that are little differentiated and biologically insignificant. That is, expression values are removed if they are within SD_COEFF×SD, where SD is the standard deviation.

Another issue is outliers, which cause a problem of expanding the distributional tails. To avoid this problem, for each column, we first compute the mean (Mean) of all expression values, and expression values, which are not within the Mean±3SD, are set at the Mean±3SD, indicating that MAX = Mean+3SD, MIN = Mean - 3SD, by which MAX_DIFF is always 6SD/BIN.

The procedure of generating frequent itemsets can be once again reviewed by using the above parameters as follows: For each experimental condition (column) of the input gene expression matrix, genes are sorted by expression values, and a window with a range of MAX_DIFF is slided over the genes in a gene-wise manner to generate so-called *items*. So then the number of items are the same as the number of genes. We can then represent the original input matrix by using the items. That is, each element of the new matrix is a set of items. Out of this matrix, we can generate a submatrix with two or more genes which share the same item for each of two or more columns (experimental conditions), and this submatrix (being an *itemset*) is exactly a bicluster with coexpressed genes under multiple conditions. So if we find frequent itemsets, which share a larger or equal number of rows than MIN_ROW, they are biclusters with a larger or equal number of genes than MIN_ROW. That is why we use frequent itemset mining for finding biclusters with coexpressed genes under multiple conditions.

### Mining maximal frequent itemsets

To reduce the redundancy of frequent itemsets, we use maximal frequent itemsets, which are those that do not have any larger frequent itemsets. SiBIC uses MAFIA [18], a software for mining maximal frequent itemsets which can output frequent itemsets keeping the number of rows (genes) larger than or equal to MIN_ROW, while the number of columns is not considered in FIM. Thus we further filter out the output of MAFIA to output only biclusters with a larger or equal number of experimental conditions than MIN_COL.

### Computing empirical 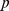-values

In order to rank biclusters and filter out nonsignificant ones, SiBIC computes empirical 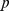-values in terms of how significantly row vectors in biclusters are correlated, as follows: For a bicluster with 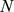 genes and 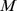 experimental conditions, matrices with the same size are randomly generated 100,000 times out of the input gene expression matrix. SiBIC then computes the following test statistic 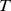 over each generated matrix:
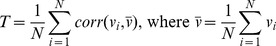
(1)where 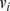 is a 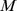-dimensional row vector of expression values and 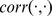 is Pearson correlation coefficient. SiBIC then computes the ratio of how many matrices among 100,000 trials have smaller 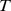 values than the 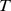 value of the bicluster we consider, resulting in the empirical 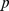-value of the bicluster. Finally SiBIC outputs the sorted biclusters with lower 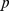-values than a certain significance level, which is 0.05 for the default setting.

### Merging biclusters

While FIM enables us to robustly enumerate all possible biclusters, it may produce too many biclusters for a user to check. In order to avoid this, we use maximal frequent itemsets, which are the largest frequent itemsets, which do not have larger frequent itemsets. However even maximal frequent itemsets also can be similar to each other. When we reduce the number of redundant biclusters without loss of the significance in coexpression, we need to have larger but still significant biclusters. SiBIC merges all biclusters, which have the same experimental conditions and partially share genes, into one bicluster, keeping the empirical 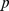-value of Eq.(1) smaller than a threshold (the default value is 0.05). We note that this is an original point of SiBIC, which has not been considered in both DeBi and BiModule.

**Figure 12:**
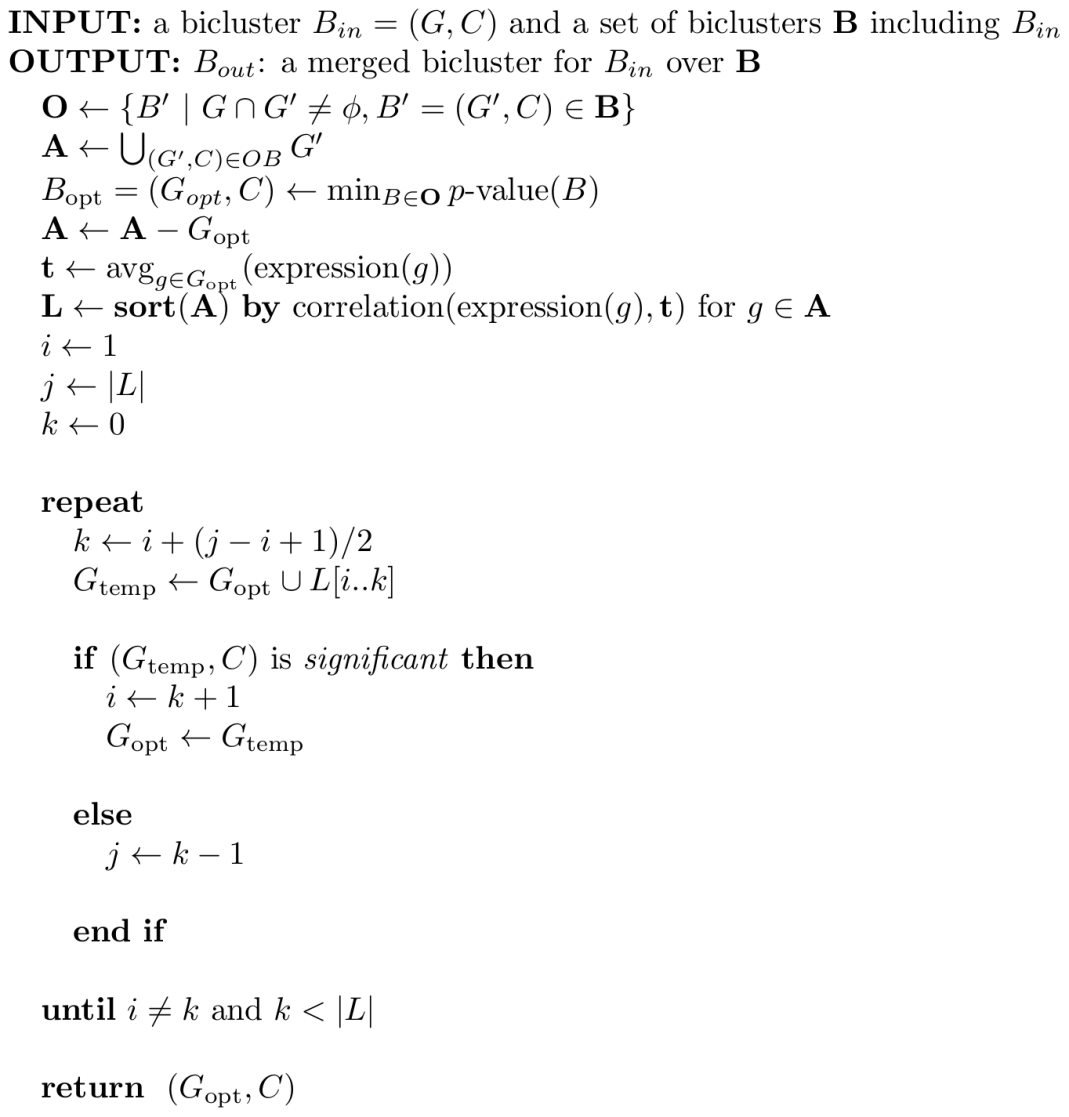
Pseudocode of merging biclusters. 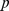
-value(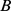) is the empirical 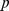-value of bicluster 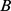, expression(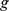) is the vector of expression values of gene 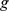, avg
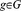
(expression(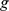)) is the vector, each value is the average of the corresponding values of vector expression (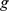) over all 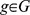, and correlation(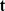, 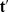) is Pearson correlation coefficient between two vectors, 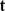 and 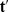.

Fig. 12 shows a pseudocode of our merging algorithm, which is based on binary search to efficiently add as many genes as possible in a small number of iterations. The inputs are a bicluster 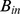 and a set of biclusters 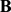 including 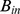. The output is a merged bicluster, 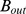, over 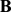. Here we write a bicluster 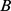 by 2-tuple 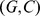, where 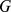 is a gene set and 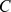 is an experimental condition set in bicluster 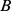. The first step of the algorithm is as follows: We generate a set of biclusters 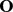, which are all overlapped with 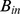, and a set of genes 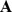 which has all genes of the biclusters in 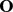. We then check the 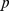-value of each bicluster of 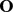 and take the bicluster with the minimum 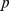-value as 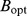, to which genes are to be added. We then, from 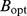, generate a vector 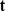 of expression values by taking the average of all row vectors of expression values in 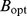. We then remove genes in 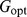 from 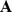, and in the main iteration of the algorithm, check to see what genes in 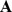 should be added.

In the main iteration, our addition of genes in 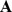 is a greedy manner in the sense that we make a list 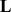 by sorting genes in 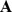 according to the (high) correlation to 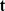 and we just think about the first part of 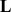 only. (The first part means, for example, the first to third genes of all ten genes in 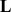.) That is, we repeat adding the first part of 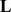 to 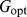 unless 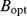 with the new 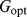 is insignificant. So now the problem is to find the first part of 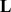, and this problem is solved by binary search in our algorithm.

### Generating gene set networks

Genes in a bicluster are supposed to have similar biological behavior. If two biclusters are overlapped, the behavior of the genes corresponding to the overlapped submatrix may be different from the other genes. Thus SiBIC represents such gene behavior difference in terms of overlapping biclusters by generating a gene set network, as follows: All genes are first decomposed into disjoint gene sets in the way that genes fall into the same set if they share the same biclusters; otherwise they are in different sets. SiBIC then generates a weighted network of gene sets, where one node is a gene set, one edge shows that the connected two nodes are in the same bicluster and the weight on an edge shows how many biclusters have the connected nodes. This means that nodes from the same bicluster generate a complete graph. We stress that a gene set network is more compact and visually more comprehensible than a gene network in which nodes are genes.

**Figure 13:**
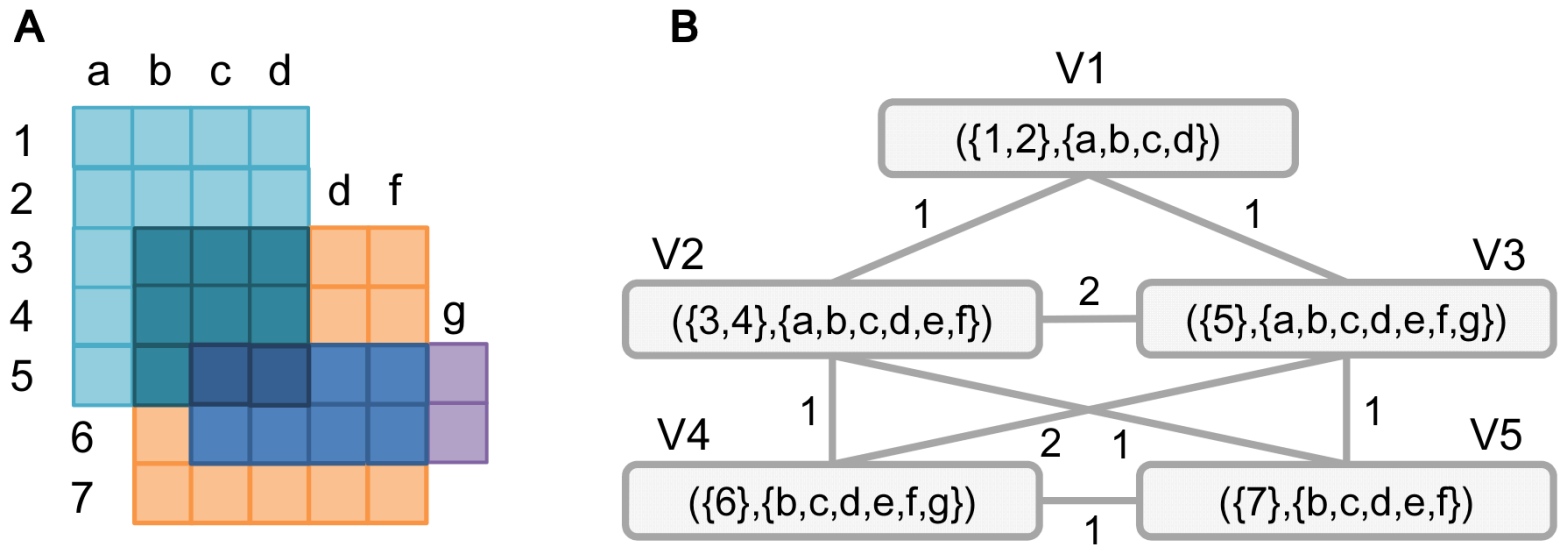
Construction of a gene set network. (A) Three overlapping biclusters: blue 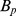, orange 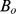 and purple 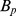 biclusters are overlapped with each other. (B) Gene set network generated from the three biclusters in (A).

Fig. 13 shows an explanatory example of the conversion from (A) overlapping biclusters to (B) a gene set network. In (A), three biclusters are overlapped with each other: a blue bicluster 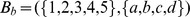, an orange bicluster 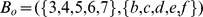 and a purple bicluster 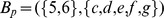. The whole genes 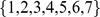 can be disjointly divided into sets 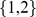, 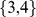, 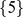, 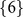 and 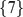, according to the biclusters having genes. Concretely speaking, gene set 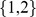 appears only in 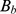, gene set 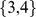 appears in 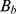 and 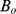, gene set 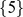 appears in all three biclusters, 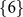 appears in 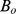 and 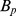, and 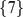 appears in 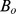. Thus each gene set has different biclusters. We can consider these sets as nodes, to be linked by edges if the corresponding gene sets are in the same biclusters. This means that one bicluster generates a complete subgraph. For instance, nodes 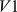, 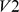 and 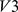 compose a complete subgraph corresponding to a blue bicluster 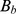. Each edge weight shows how many biclusters share the nodes connected by the corresponding edge. For example, edge 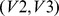 is weighted by two because 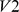 and 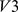 are shared by two biclusters 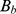 and 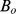.

As shown in Fig. 13 (B), nodes have experimental conditions of biclusters, indicating that each node also represents one bicluster with an expression pattern over the experimental conditions of the input biclusters.

### Graphical user interface of gene set networks

**Figure 14:**
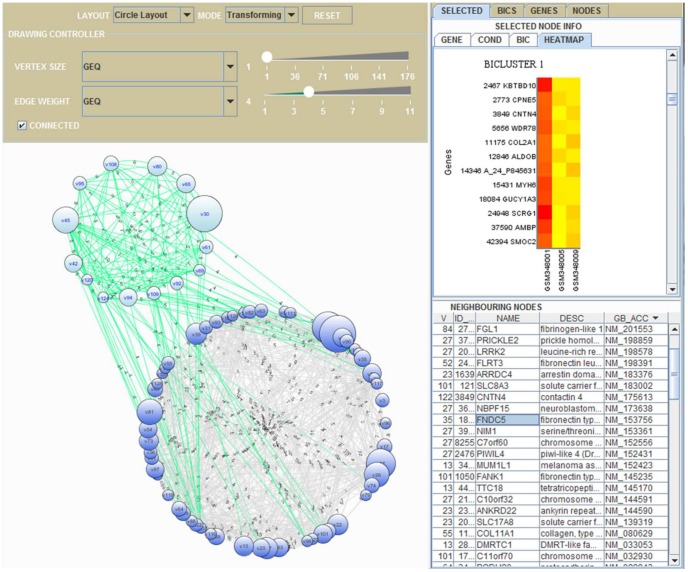
Graphical user interface of a gene set network (See the main text for details).

Fig. 14 shows an example of graphical user interface of SiBIC, by which users can manipulate gene set networks, i.e. the final results of SiBIC. The GUI is a Java applet which can be obtained from the result page of SiBIC and run in a web browser. We developed the GUI using Java 6 Swing and JUNG (Java Universal Network/Graph Framework) library 2.0. As shown in Fig. 14, the GUI has the left and right panes. The GUI allows users to conduct GO term enrichment analysis in a more flexible manner than the case of using only a single bicluster. For example, a user can pick up genes in the node with the maximum degree and its neighboring nodes or genes in the most significant bicluster. After selecting genes, users can run DAVID, a third-party tool for GO term enrichment analysis. We here explain the left and right panes more in detail.

Left pane: The left pane has the drawing controller on the top and the network viewer on the bottom. In the top, a user can select the network ‘LAYOUT’ out of five types: ‘Circle’, ‘KK’, ‘FR’, ‘Spring’ and ‘ISOM’. A user can further choose ‘MODE’ of the network viewer, where ‘Picking’ allows a user to pick and drag the vertices of interest, while ‘Transforming’ enables a user to drag the whole network by using a mouse. A user can filter out subnetworks by ‘VERTEX SIZE’ or ‘EDGE WEIGHT’. By unchecking the ‘CONNECTED’ box in the top, the network viewer can show unconnected networks. Clicking a vertex under the ‘Picking’ mode updates the information of the right pane. A helpful function of this side is that a user can choose (click) multiple nodes at the same time by dragging a mouse to make a rectangle so that it encompasses the multiple nodes and clicking one of the selected nodes.

Right pane: The right pane has four tabs: ‘SELECTED’, ‘BICS’, ‘GENES’ and ‘NODES’. The ‘SELECTED’ tab shows the information on the selected nodes in the left pane, where a lot of features such as gene names, experimental conditions, and heat maps are shown. The other three tabs, ‘BICS’, ‘GENES’ and ‘VERTICES’ provide the information on the entire network in the left pane. The ‘BICS’ tab shows a table of all biclusters of the network. The ‘GENES’ tab shows a table of all genes in all nodes in the network. The ‘NODES’ tab shows a table of features of all nodes, such as the degree and weighted degree.
